# Prostatic-Like Syndrome in a Woman with Chronic Lymphocytic Leukemia: Sequential Kinase Inhibitor Therapy

**DOI:** 10.1155/2017/3869020

**Published:** 2017-07-02

**Authors:** Diego Velasco-Rodríguez, Miguel Piris-Villaespesa, Carmen Soteras, Ana Vallés, José Antonio García-Marco, José Antonio García-Vela

**Affiliations:** ^1^Department of Hematology, Fundación Jiménez Díaz, Madrid, Spain; ^2^Department of Hematology, Hospital Universitario Ramón y Cajal, Madrid, Spain; ^3^Department of Radiology, Hospital Universitario Ramón y Cajal, Madrid, Spain; ^4^Department of Hematology, Hospital Universitario Puerta de Hierro, Madrid, Spain; ^5^Department of Hematology, Hospital Universitario de Getafe, Madrid, Spain

## Abstract

Chronic lymphocytic leukemia (CLL) is an incurable lymphoproliferative disorder with a heterogeneous genetic and clinical course. Two kinase inhibitors, ibrutinib and idelalisib, have demonstrated achievement of complete and durable remissions in relapse/refractory genetically unselected CLL patients. We present a case of relapsed CLL with extensive disease and hourglass deformity of urinary bladder as a result of the compression of two extraperitoneal paravesical soft tissue bulky masses, with excellent response to sequential kinase inhibitor therapy.

## 1. Introduction

Chronic lymphocytic leukemia (CLL) is an incurable lymphoproliferative disorder with a heterogeneous genetic and clinical course. When treatment is indicated according to the IWCLL criteria [1], young fit patients without p53 abnormalities should be treated with chemoimmunotherapy, and the combination of FCR is the best option for patients under 65 years old [2]. In patients with a short progression free survival or refractory to chemoimmunotherapy, either the combination of idelalisib plus rituximab or ibrutinib monotherapy is the treatment of choice. P53 disruption is one of the most important prognostic and predictive factors in the clinical evaluation of CLL patients.

Deletion of chromosome 17p13 region, which contains the p53 gene locus, and mutation of* TP53* gene should be analysed in all CLL patients before starting treatment. Both ibrutinib and idelalisib plus rituximab have demonstrated high activity and achievement of durable remissions in relapse/refractory genetically unselected CLL patients. The most common reason for discontinuation of these two kinase inhibitors (KI) is toxicity. Immune diarrhea, transaminitis, and opportunistic infections are the most frequent side effects of idelalisib. Ibrutinib has been related to an increase in bleeding and atrial fibrillation. A feasible option in this context is changing to the other KI with acceptable results.

## 2. Case Report

A 62-year-old woman with CLL Binet stage B of 8 years duration was referred to our institution with disease progression. She had been previously treated with FCR (fludarabine, cyclophosphamide, and rituximab) as first line treatment and also with bendamustine plus rituximab and CHOP-R (cyclophosphamide, doxorubicin, vincristine, prednisolone, and rituximab) in successive relapses. She presented with asthenia, fever, and back pain and had difficulty starting urination with a feeling of incomplete bladder emptying for the last month. In the physical exam she had bulky axillary masses, splenomegaly, and a painful pelvic mass.

**Figure 1:**
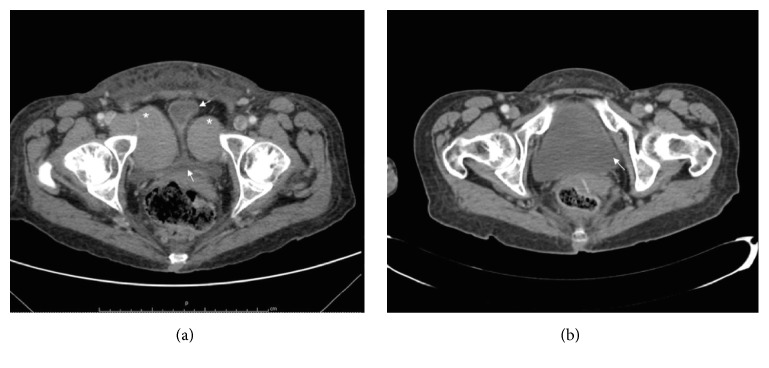
Pelvic CT before treatment with rituximab plus idelalisib (a). Hourglass deformity of urinary bladder (arrows) was seen as a result of the compression of two extraperitoneal paravesical soft tissue bulky masses (asterisks). After three months of idelalisib (b) a complete resolution of the masses was observer with a normal bladder.

Her peripheral blood showed lymphocytosis (52.9 × 10^9^/l) with normal hemoglobin concentration and platelet count and elevated lactate dehydrogenase (546 iu/l) (140–240). A May-Grünwald-Giemsa-stained peripheral blood film revealed typical small mature lymphocytes with condensed chromatin, with less than 10% of prolymphocytes. A typical CLL phenotype was found with 80% of B cells CD19+ with coexpression of CD5, CD23, CD200, and lambda light chain restriction and weak expression of CD20, CD22, and CD79b. Computerized tomography (CT) scanning of the thorax, abdomen, and pelvis demonstrated extensive bulky axillary, mediastinal, retroperitoneal, inguinal, and pelvic lymphadenopathies. Hourglass deformity of urinary bladder (arrows) was seen as a result of the compression of two extraperitoneal paravesical soft tissue bulky masses (asterisks) (Figure 1(a)).

Pathologic features in an axillary lymph node biopsy were consistent with CLL, with no evidence of Richter transformation (RT).* IGH* genes were not mutated and fluorescence in situ hybridization (FISH) was negative for chromosome 12 gains and also for deletions of 13q14, 11q22-23, 6q, and 17p13. Mutation of* TP53* gene was demonstrated by Sanger sequencing.

Treatment with idelalisib (150 mg twice daily) and rituximab was started, achieving a very good partial response with lymphocytosis after three months. Complete recovery of urinary function was observed and a repeat CT showed disappearance of the masses and a normal bladder (arrow, right) (Figure 1(b)). 10 months later the patient began with diarrhea grades 3-4 with normal stool analysis including cytomegalovirus investigation. Idelalisib was stopped and the patient was treated with oral beclometasone 5 mg daily and loperamide. After three weeks of symptomatic treatment, diarrhea disappeared and idelalisib was reinitiated at a lower dose (100 mg twice daily) with recurrence of the diarrhea 2 week later. Idelalisib was definitely withdrawn and replaced by ibrutinib (420 mg daily). At the present time, the patient remains in partial remission after 24 months of follow-up without recurrence of the urinary symptoms or progression with sequential kinase inhibitor therapy with idelalisib and ibrutinib due to intolerance to the former.

## 3. Discussion

At diagnosis, the incidence of p53 abnormalities is low and has been reported to be 4–8% in patients with CLL. As disease progresses, the incidence rises to 10–12% at the time of first line treatment, 40% in fludarabine-refractory cases, and 50–60% in Richter syndrome. Mutations represent the most frequent form of TP53 inactivation in CLL and are frequently (70% of the cases) accompanied by the loss of the second allele (17p13 deletion).

The frequency of mutations lacking 17p13 deletion is variable, but in general they represent 30% of all TP53 defects, whereas sole 17p13 deletion with the absence of* TP53* mutation is less frequent (10% of all* TP53* defects) [3]. The clinical implication of these molecular observations is that, in order to perform a correct evaluation of the* TP53* gene status in CLL, it is recommended to assess both the presence of chromosome 17p13 deletion by FISH and of* TP53* mutations by Sanger gene sequencing [4]. FISH analysis of our case did not show 17p13 deletion but* TP53* mutation was detected by Sanger.

An axillary lymph node biopsy was performed to rule out histologic transformation. RT of CLL occurs in about 5–10%, typically into diffuse large B cell lymphoma. RT carries a very poor prognosis and should be suspected when a CLL patient develops sudden onset of B symptoms, accompanied by enlarging lymph nodes. Laboratory studies commonly associated with RT include an important elevation of lactate dehydrogenase [5].

The Bruton tyrosine kinase (BTK) inhibitor ibrutinib and the phosphoinositide 3-kinase *δ* (PI3K*δ*) inhibitor idelalisib have demonstrated high activity and achievement of durable remissions in relapse/refractory genetically unselected CLL patients [6].

In the study 116, idelalisib in combination with rituximab showed a significant improvement in both PFS and overall survival (OS) in relapsed CLL patients with increased number of comorbidities (45% of whom had del17p13) [7]. The efficacy of idelalisib plus rituximab appeared not to be affected by the presence of del17p13/P53 mutation in a preliminary, retrospective subgroup analysis of this study, indicating that this combination is a valid treatment option for this high risk group. Our patient was treated with idelalisib plus rituximab and achieved a partial response three months after starting treatment. A CT showed a complete resolution of the axillary and pelvic masses and the urinary symptoms disappeared. However, 10 months later the patient experienced a colitis grade 3 that was initially responsive to drug hold and oral beclometasone. Idelalisib was restarted at a lower dose (100 mg twice daily) with recurrence of the diarrhea, so it was finally stopped. Delayed colitis occurring at a median of 7 months after starting treatment is seen in 14% of patients. Recent data have demonstrated CD8+ T lymphocytes in the colon, and an immunological mechanism of toxicity has been postulated. PI3K*δ* is critical in the survival and function of regulatory T lymphocytes and hepatotoxicity is associated with a decrease in T regulatory T cells 1 month after starting idelalisib [8].

Treatments with an alternative KI or venetoclax therapy appear to be superior to chemoimmunotherapy combinations when failure to KI is observed [9].

Ibrutinib [10] was therefore started (420 mg/day) and the patient is still in partial remission after 24 months of follow-up. A recent multicentric retrospective analysis on 178 patients with CLL who discontinued KI therapy showed that the most common reason for discontinuation was toxicity (51%). Median PFS in KI intolerant patients treated with an alternative KI was not reached versus 7 months for patients with CLL progression [11]. Recent evidence suggests that ibrutinib might be superior to idelalisib as first KI [9].

Venetoclax (an anti-Bcl2 therapy) is the best alternative in CLL patients who fail to KI [12]. In a recent study, it has been suggested that, in the setting of KI failure, the use of venetoclax upon ibrutinib failure might be superior to idelalisib [9].
